# Pixelated Carrier Phase-Shifting Shearography Using Spatiotemporal Low-Pass Filtering Algorithm

**DOI:** 10.3390/s19235185

**Published:** 2019-11-26

**Authors:** Peizheng Yan, Xiangwei Liu, Shuangle Wu, Fangyuan Sun, Qihan Zhao, Yonghong Wang

**Affiliations:** School of Instrument Science and Opto-Electronics Engineering, Hefei University of Technology, Hefei 230009, China; pzyan@hfut.edu.cn (P.Y.); hfutlxw@163.com (X.L.); wslhfut@126.com (S.W.); laderniere@126.com (F.S.); zhaoqihan@mail.hfut.edu.cn (Q.Z.)

**Keywords:** shearography, non-destructive testing, phase measurement, pixelated carrier phase-shifting, spatiotemporal low-pass filtering

## Abstract

Shearography has been widely used in non-destructive testing due to its advantages in providing full-field, high precision, real-time measurement. The study presents a pixelated carrier phase-shifting shearography using a pixelated micropolarizer array. Based on the shearography, a series of shearograms are captured and phase maps corresponding to deformation are measured dynamically and continuously. Using the proposed spatiotemporal filtering algorithm in the complex domain, the set of phase maps are simultaneously low-pass filtered in the spatial and temporal domains, resulting in better phase quality than spatial low-pass filtering. By accumulating the temporally adjacent phase, the phase corresponding to large deformation can be evaluated; thus, large deformations can be accurately measured and protected from speckle noise, allowing internal defects to be easily identified. The capability of the proposed shearography is described by theoretical discussions and experiments.

## 1. Introduction

The internal defects of a workpiece can reduce its mechanical properties and even cause serious consequences [1]. Non-destructive testing (NDT) is the ideal approach for detecting defects and structural issues in a non-invasive way. Shearography [2,3] is an interferometric technique for deformation measurement of rough object surfaces that can reveal the internal defects of an object by identifying its defect-induced deformation anomalies under stress [4,5]. It has been widely used in NDT due to its advantages in providing full-field, high precision, real-time measurement [6,7,8,9,10]. Notably, the US Federal Aviation Administration (FAA) has endorsed shearography for inspecting aircraft tires [11].

The phase-shifting technique is key to extracting the interferometric phase of shearograms. It can be divided into temporal and spatial carrier phase-shifting techniques. The temporal phase-shifting technique evaluates the phase from multiple shearograms captured sequentially [12,13], offering the advantages of precise measurement and ease of phase calculation, but requiring the measured objects to remain stationary during the measurement process. Therefore, the temporal phase-shifting method performs well for static or quasi-static measurements. In contrast, the spatial carrier technique allows phase to be evaluated from a single shearogram, which allows dynamic measurement, a feature that has made the spatial carrier technique a focus of research interest [14,15].

In the spatial carrier phase-shifting technique, some particular optical structures are designed that a spatially dependent carrier phase is added to the wavefront phase. Several methods of spatial phase-shifting have been developed for shearography. In two spatial phase-shifting shearography techniques—Mach–Zehnder interferometer-based [14,16] and Michelson interferometer-based [5,17]—the spatial carrier frequency is generated by the tilt angle between the two sheared images. However, both carrier frequency and shearing amount are introduced by the tilting angle, with the result that these shearography techniques can work only within certain thresholds of shearing [18]. A carrier frequency can also be generated by using multiple apertures and adjusting their distances, which allows independent adjustment of shearing amount and spatial carrier frequency [18,19,20,21,22]. For the above spatial carrier shearography, the carrier phase is linear. The phase can be calculated from the complex amplitude by applying a properly selected windowed inverse FT (WIFT), which requires the holos in the Fourier spectrum are isolated from each other. To generate non-overlapping halos in Fourier spectrum, it is important to produce a high enough carrier frequency and a small enough aperture, which leads to a large speckle size and speckle noise and decreases the quality of the evaluated phase.

Another spatial carrier phase-shifting technique uses pixelated periodic carrier instead of linear carrier phase. The pixelated carrier technique encodes four phase-steps into a single shearogram using a pixelated micropolarizer array physically attached to the image sensor [23,24]. This method is compact and easy to realize component alignment. The pixelated carrier phase-shifting interferometry has a higher spatial frequency response than a linear carrier phase-shifting interferometry [25], which has been used in a variety of commercial interferometers to measure the shape of smooth surfaces. However, it still suffers from speckle noise when applied to shearography [26], and the quality of the phase evaluation is lower than that of temporal phase-shifting method. Low-pass phase filtering is required to obtain a good phase map due to the speckle noise [27]. Conventional filtering method values are performed for a single-phase map. With more filtering times and a larger filtering window, the filtered phase map will be smoother, but details will be lost. It is especially difficult to achieve a good filtering phase map when the deformation is large, and the speckle particle size is larger than the spacing of interference fringes.

Beside the temporal and spatial carrier phase-shifting techniques, spatiotemporal phase-shifting and analysis methods have been applied to interferometer [28,29] and fringe projection profilometry [30], which make use of the specific characteristic in the spatial and temporal domains. In this research, the shearograms with speckle noise are low-pass filtering based on the smoothing properties of deformation in both domains. A pixelated carrier phase-shifting shearography using a Michelson interferometer is proposed. The complex image containing the information of phase map is captured continuously by the pixelated carrier phase-shifting shearograph. Then, the complex image is low-pass filtered in the spatial and temporal domains simultaneously. After low-pass filtering, the phase map is evaluated from the complex image, and a smoothed phase is accumulated to obtain the phase map of large deformation.

## 2. Methods

**Figure 1:**
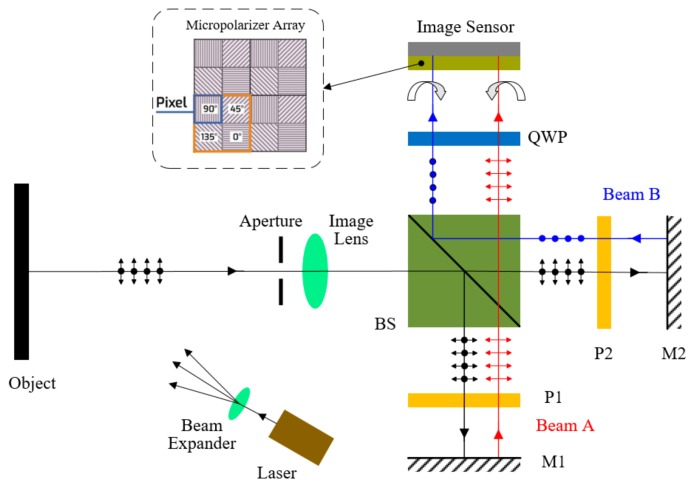
Schematic diagram of the pixelated carrier phase-shifting shearography.

Pixelated carrier phase-shifting shearography using a Michelson interferometer is shown in Figure 1. A laser beam is expanded to illuminate the object. Because the surface is rough, laser scattered from it has random polarization. The object beam is divided into two beams, A and B, by the beam splitter (BS), which pass through the polarizers (P1 and P2), and are reflected by mirrors (M1 and M2), respectively. The extinction ratio of polarizers P1 and P2 is 500:1. The shear between the images can be adjusted by tilting one or both mirrors. When the two beams are once again combined by the BS, they have linear polarizations orthogonal to each other, determined by setting the polarization direction of P1 and P2 orthogonal to each other. The angle between the optical axis of the quarter-wave plate (QWP) and the polarization directions of the beam A or the beam B are both 45 degrees. Thus, the two linearly polarized beams passed through the QWP are converted into right- and left-handed circularly polarized light, respectively. Then, the beams pass through the micropolarizer array and interfere at the image sensor. In the micropolarizer array, a set of four (2 × 2) micropolarizers with 0°, 45°, 90°, and, 135° polarization orientations are arranged into a “unit cell” that repeats continuously over the entire array. The micropolarizer array is physically attached to the camera, and every micropolarizer is aligned with a single pixel. Because right- and left-handed circularly polarized beams subject to interference by a linear polarizer have a phase shift proportional to twice the rotation angle of the polarizer, the micropolarizer array adds a phase shift of 0°, 90°, 180°, and 270° between beam A and beam B. The shearogram on the imaging plane can be expressed as

(1) $$I\left(x,y\right)=a\left(x,y\right)+b\left(x,y\right)\cos\left[\Delta \varphi \left(x,y\right)+\phi_{c}\left(x,y\right)\right]$$

where *a*(*x*,*y*) and *b*(*x*,*y*) are the background illumination and the amplitude of the fringe pattern, respectively, and $\Delta \varphi \left(x,y\right)=\varphi \left(x+\Delta x,y\right)-\varphi \left(x,y\right)$ are the phase difference of beams A and B. For simplicity, the shearing direction is supposed to be in the x-axis and Δx is the shear amount. Then, $\phi_{c}\left(x,y\right)$ is the phase shift introduced by the pixelated micropolarizer array, which has the following form:

(2) $$\begin{array}{ll} \phi_{c}\left(2m,2n\right)=0, & \;\;\;\;\;\phi_{c}\left(2m+1,2n\right)=\frac{3\pi }{2}\\ \phi_{c}\left(2m,2n+1\right)=\frac{\pi }{2}, & \;\;\;\;\;\phi_{c}\left(2m+1,2n+1\right)=\pi \end{array}$$

where *m* and *n* are pixel coordinates.

**Figure 2:**
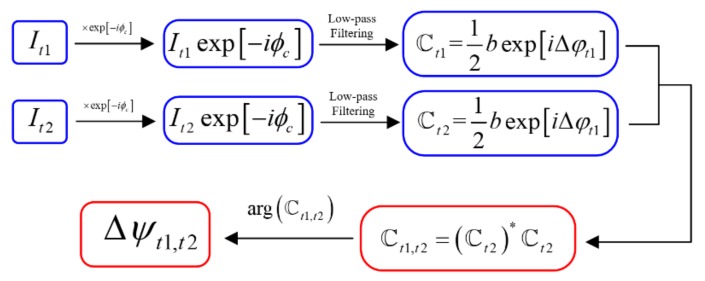
Flow chart of the pixelated carrier phase-shifting algorithm.

The flow chart of the algorithm to pre-process the shearogram is shown in Figure 2.

The shearogram is multiplied by the complex carrier $\exp\left[-i\phi_{c}\left(x,y\right)\right]$, resulting in Equation (3):

(3) $$\begin{array}{ll} I\exp\left[-i\phi_{c}\right]= & a\exp\left[-i\phi_{c}\right]\\ & +\frac{1}{2}b\exp\left[-i\left(\Delta \varphi +2\phi_{c}\right)\right]\\ & +\frac{1}{2}b\exp\left[i\Delta \varphi \right] \end{array}$$

where the coordinates $\left(x,y\right)$ in the formula are omitted for simplicity.

The Fourier transform of the complex product obeys the following law: $\frac{1}{2}b\exp\left[i\Delta \varphi \right]$ is located at the low-frequency part of the frequency spectrum, and other terms of Equation (3) are located at the high-frequency part. If the aperture of the Michelson interferometer is small enough, $\frac{1}{2}b\exp\left[i\Delta \varphi \right]$ can be separated by a low-pass filter [31,32]. Label the complex image $\frac{1}{2}b\exp\left[i\Delta \varphi \right]$ as ℂ, the angle of which is the phase map Δφ. The phase map $\Delta \varphi_{t}$ at time *t* can be determined from the corresponding complex image ${\mathbb{C}}_{t}$. Then, ${\mathbb{C}}_{t1,t2}$ is defined as in Equation (4):

(4) $${\mathbb{C}}_{t1,t2}={\left({\mathbb{C}}_{t2}\right)}^{*}{\mathbb{C}}_{t2}=\frac{1}{4}b_{t1}b_{t2}\exp\left[i\left(\Delta \varphi_{t2}-\Delta \varphi_{t1}\right)\right]$$

The angle of ${\mathbb{C}}_{t1,t2}$ is the phase difference of two times, which is proportional to the first derivative of out-of-plane deformation, as shown in Equation (5):

(5) $$\Delta \psi_{t1,t2}=\Delta \varphi_{t2}-\Delta \varphi_{t1}=\frac{4\pi }{\lambda }\frac{\partial w\left(x,y\right)}{\partial x}\Delta x$$

The defect of the object can be detected by judging the anomalies in the phase map $\Delta \psi_{t1,t2}$.

**Figure 3:**
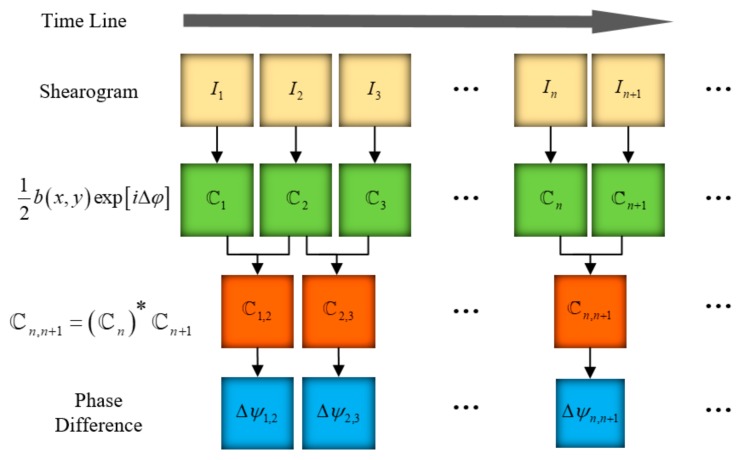
Processing of the time series shearograms.

The phase map $\Delta \psi_{t1,t2}$ evaluated according to the above algorithm suffers from severe speckle noise. Speckle noise causes the quality of the phase map to decrease, and affects the identification of defects; thus, phase filtering is important for shearography. The spatial low-pass filtering method is carried out for a single-phase diagram, which uses the low-frequency characteristics of the deformation over the spatial domain. When the deformation is too large, high-frequency component of deformation increases, making it difficult to achieve a good filtering result. By the pixelated carrier phase-shifting shearography, a series of shearograms $I_{n}$ (n=1, 2, ⋯, N) are continuously captured in a manner synchronized with the camera’s frame rate, as shown in Figure 3. The subscript n is the serial number of the continuous acquisition shearogram, which has the same meaning as the subscripts t1 and t2 in Equation (4). Every two temporally adjacent shearograms can be used to calculate the complex image ${\mathbb{C}}_{n,n+1}$ and the phase map $\Delta \psi_{n,n+1}$. The deformation of the same point on the surface changes with low frequency over time. Using this feature, the phase of a special pixel can also be filtered in the time domain. 

**Figure 4:**
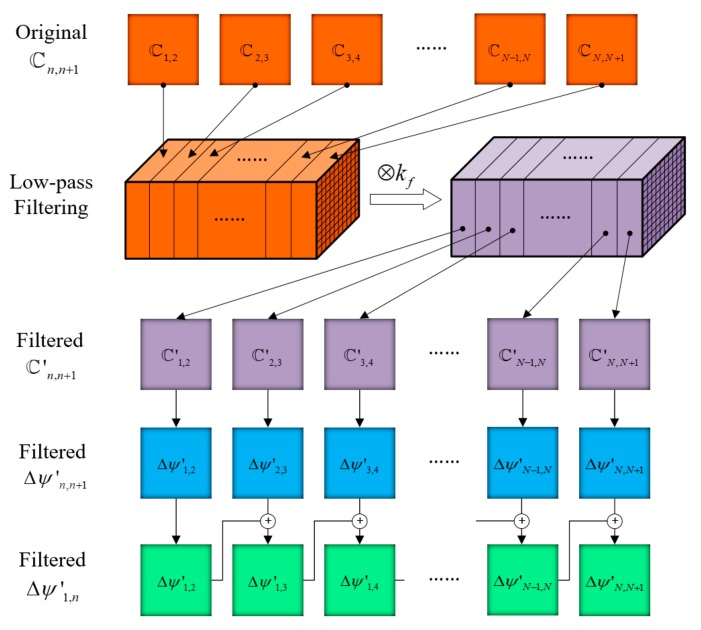
The spatiotemporal low-pass filtering process of time series shearograms.

Because the phase map $\Delta \psi_{n,n+1}$ is wrapped to 2π, discontinuous jumps of phase will appear, which will lead to errors of filtering the phase over time and spatial domain. To solve this problem, the complex image ${\mathbb{C}}_{n,n+1}$ is first low-pass filtered before the smoothed phase is determined from the filtered complex image. To make full use of the low-frequency characteristics of phase, the complex images ${\mathbb{C}}_{n,n+1}$ are simultaneously low-pass filtered in the time and spatial domains. The outline of the spatiotemporal low-pass filtering process is shown in Figure 4.

All of the resulting 2D complex images ${\mathbb{C}}_{n,n+1}$ are sequentially recomposed into a 3D matrix, the third dimension of which is the time domain. The constructed 3D complex matrix is then low-pass filtered by convolution with a low-pass filter kernel in the spatial and temporal domain. The low-pass filter kernel is a 3D matrix. For example, a 3D kernel ($k_{f}$) with the dimension size of M*M*T, all elements of which have a value of 1/(M*M*T), is a low-pass average filter. Other types of low-pass filters, such as Gaussian filters, can also be used. After low-pass filtering in the spatial and temporal domains, the constructed 3D complex matrix is decomposed into a series of 2D complex images, ${\mathbb{C}}_{n,+1}^{\prime }$. Then, the smoothed phase maps $\Delta \psi_{n,n+1}^{\prime }$ can be determined by calculating the angle of ${\mathbb{C}}_{n,n+1}^{\prime }$, which can be expressed by Equation (6):

(6) $$\Delta \psi_{n,n+1}^{\prime }=\arg\left({\mathbb{C}}_{n,n+1}^{\prime }\right)$$

The smoothed phase maps $\Delta \psi_{n,n+1}^{\prime }$ is the phase difference of two temporally adjacent shearograms, and can be expressed using Equation (7):

(7) $$\Delta \psi_{c,c+1}^{\prime }=\Delta \varphi_{c+1}-\Delta \varphi_{c}$$

Thus, by accumulating the temporally adjacent phase $\Delta \psi_{n,n+1}^{\prime }$, the phase corresponding to deformation over a long time can be evaluated using Equation (8), which allows determination of deformation at any time with reference to any other time:

(8) $$\Delta \psi_{1,N}^{\prime }=\Delta \varphi_{N}-\Delta \varphi_{1}=\sum_{c=1}^{N-1}\Delta \psi_{c,c+1}^{\prime }$$

In next part, experimental data will show the advantages of the filtering algorithm compared with spatial low-pass filtering methods.

## 3. Experiment

**Figure 5:**
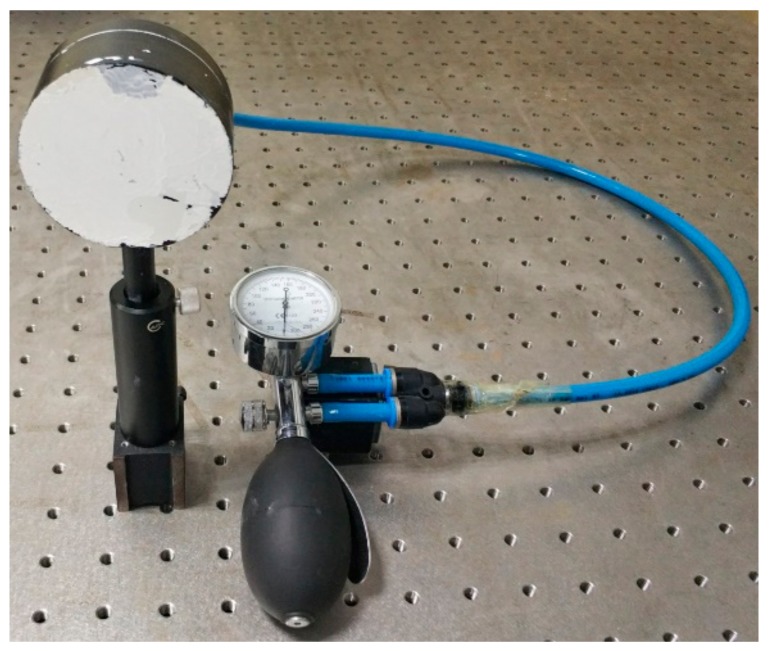
The object capable of deforming along with pressure change.

Experiments have been designed to verify the pixelated carrier phase-shifting shearography and the spatiotemporal filtering algorithm in the complex domain. The object under test is a hollow cylinder with multiple internal defects, as shown in Figure 5. The diameter of the surface is 70 mm. The surface coating of the test object is partially dropped, but the surface remains rough to generate speckle interference. The focal length of the image lens used in the experiment is 25mm. By increasing the air pressure in the hollow position and letting the air release, the surface of the object will be deformed continuously as the air pressure decreases. A 532-nm laser (Changchun New Industries Optoelectronics Technology Co., Ltd., Changchun, China, continuous wave, 200 mW) is used as the laser source. The image sensor with a micropolarizer array attached to it is the polarization CMOS image sensor (IMX250MZR) from Sony (Tokyo, Japan), which has a resolution of 2464 × 2056 pixels. 

**Figure 6:**
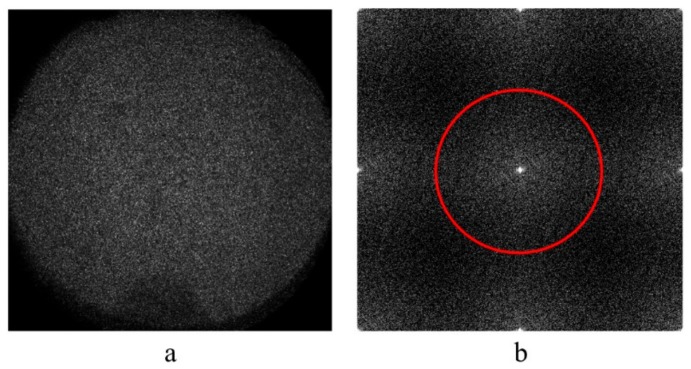
The shearogram (**a**) and frequency spectrum of a shearogram (**b**).

Figure 6 shows a shearogram and frequency spectrum of $I\left(x,y\right)\exp\left[-i\phi_{c}\left(x,y\right)\right]$. The spectrum of $\frac{1}{2}b\left(x,y\right)\exp\left[i\Delta \varphi \right]$ marked by a red circle is located at low frequencies and separated from other terms, so can be extracted by a low-pass filtering process shown in Figure 2. 

**Figure 7:**
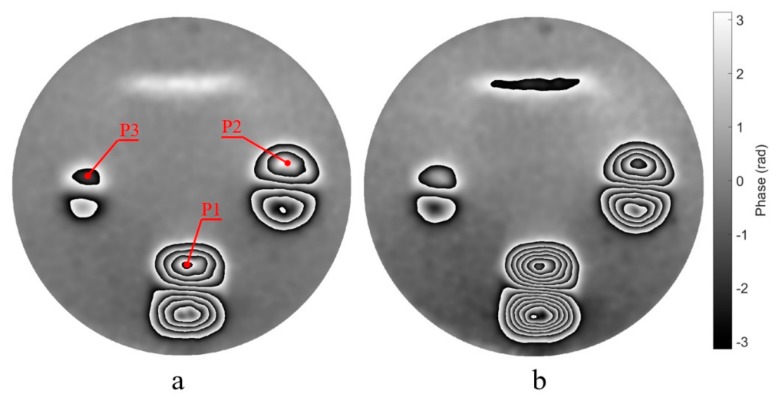
The phase difference maps at 3s (**a**) and 6s (**b**) respect to 0s.

During deformation of the surface, 120 shearograms $I_{n}$ (*n* = 1, 2, …, 120) are continuously captured over 6 s with the frame rate of 20 fps. The spatiotemporal filtering algorithm in the complex domain is applied to these shearograms. The low-pass filter kernel is an average filter with a window size of 11 × 11 × 5 and the number of filtering is 10. After filtering, the smoothed phase difference maps $\Delta \psi_{n,n+1}^{\prime }$ (*n* = 1, 2, …, 119) of every two temporally adjacent shearograms are obtained. Then, 119 phase maps, $\Delta \psi_{1,n}^{\prime }$ (*n* = 2, …, 120), corresponding to the deformation at any time with respect to the start time are obtained using Equation (8). These 119 phase maps are synthesized into a video (see the Supplementary Materials). The phase maps corresponding to the deformation at 3 s and 6 s are shown in Figure 7, demonstrating the good filtering effect. Four internal defects can easily be identified in the workpiece in Figure 7. The phase maps shown in Figure 7 and the video are wrapped to exhibit four defects clearly.

**Figure 8:**
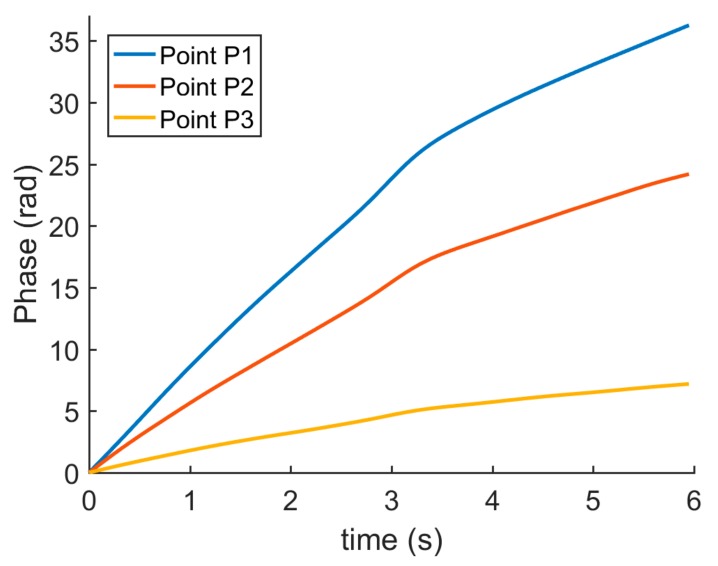
The phase change three specific pixels over time.

The following is a detailed analysis of the filtering result. The curves of the phase change of three specific pixels marked in Figure 7 over time is shown in Figure 8. Because of the temporal low-pass filtering process, the curves are smooth.

The smoothed phase difference map of two temporally adjacent shearograms, for example $I_{10}$ and $I_{11}$, is calculated using the spatiotemporal filtering algorithm and the spatial filtering algorithm respectively. The filter kernel of the spatiotemporal filtering algorithm is an mean filter with a window size of 11 × 11 × 5, where 11 × 11 is in the spatial dimension and 5 is in the temporal dimension. The filter kernel of the spatial filtering algorithm is an mean filter with a window size of 11*11, which has the same spatial size with the spatiotemporal kernel. The filtering numbers of both algorithms are 10.

**Figure 9:**
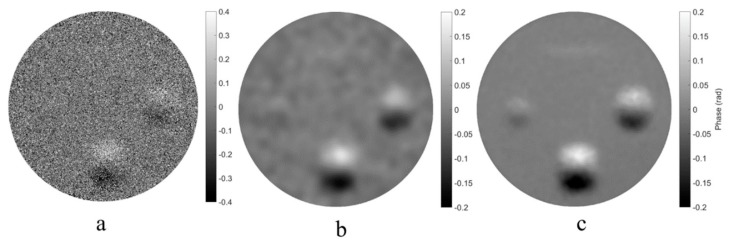
(**a**) the original phase map $\Delta \psi_{10,11}$ (**b**) smoothed phase map by spatial low-pass filtering, (**c**) smoothed phase map by spatiotemporal low-pass filtering.

The results of applying the spatial and the spatiotemporal low-pass filtering algorithms are shown in Figure 9b and c, respectively. The upper and left defect can be identified from Figure 9c more easily and clearly than from Figure 9b.

This comparison shows that for the same filter window size and number of filtering numbers, the spatiotemporal filtering algorithm achieves a less noisy phase than the spatial filtering algorithm.

**Figure 10:**
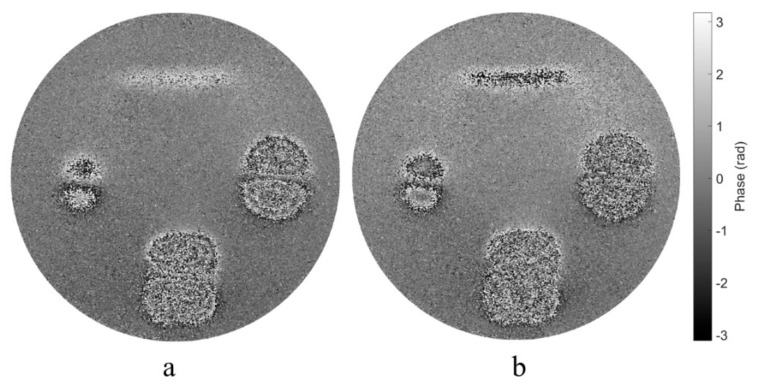
Original phase difference map at 2s (**a**) and 3s (**b**) respect to 0s calculated using Equation (4).

To obtain the phase difference of two shearograms over a long time corresponding to large deformation, traditional method is using Equation (4) to calculate it directly from the two shearograms. Figure 10 shows the results of the method. The phase maps in Figure 10 are unable to conduct low-pass filtering, because in the lower area and the right area, the interferometric fringe is totally mixed with speckle noise and impossible to distinguish. Such a large deformation has a high frequency, which increases the difficulty of filtering or even leads to filtering failure. However, by accumulating the temporally adjacent phase based on the spatiotemporal filtering algorithm, the phase corresponding to large deformation over a long period of time can be evaluated with high quality, as shown in Figure 7.

## 4. Discussion

This work presents a method for pixelated carrier phase-shifting shearography using a Michelson interferometer. A periodic pixelated carrier phase shift is added into the shearograms by a pixelated micropolarizer array physically attached to the image sensor. Based on the proposed interferometer, the relay optical lens can be used to increase the angle of view. The pixelated carrier phase-shifting technique can also be applied to the shearography based on Mach–Zehnder interferometer.

The original phase map can be calculated from the shearogram via Fourier transform. Then, a spatiotemporal low-pass filtering algorithm in the complex domain is used to process the shearograms. The use of a spatiotemporal filtering algorithm in the complex domain has several advantages: First, since the phase difference between two temporally adjacent shearograms is relatively small and the high-frequency components are limited, low-pass filtering is easy to implement. Second, both low-frequency characteristics in spatial and temporal domain are used. Third, filtering in the complex domain can avoid the effects of discontinuous phase-wrapping-induced jumps on low-pass filtering. Fourth, large deformations over a long time can be accurately measured and protected from speckle noise. A drawback of the spatiotemporal low-pass filtering algorithm is a big data storage, because a series of shearograms need to be recorded and analyzed when using the algorithm.

Based on the pixelated carrier phase-shifting shearography and the spatiotemporal filtering algorithm, deformation of an object over time under load can be measured continuously with good quality, providing data that allow internal defects to be easily identified.

## Supplementary Materials

The following are available online at https://www.mdpi.com/1424-8220/19/23/5185/s1, Video S1: Phase variation with time.avi.
